# Role of Hypovitaminosis D in the Pathogenesis of Obesity-Induced Insulin Resistance

**DOI:** 10.3390/nu11071506

**Published:** 2019-07-01

**Authors:** Emanuela A. Greco, Andrea Lenzi, Silvia Migliaccio

**Affiliations:** 1 Department of Experimental Medicine, Section of Physiopathology, Endocrinology and Food Science, University of Rome Sapienza, 00161 Rome, Italy; 2 Department of Movement, Human and Health Sciences, Section of Health Science, University of Rome Foro Italico, 00135 Rome, Italy

**Keywords:** obesity, insulin resistance, type 2 diabetes, vitamin D, hypovitaminosis D, inflammation

## Abstract

Obesity and type 2 diabetes have both rapidly increased during the last decades and are continuing to increase at an alarming rate worldwide. Obesity and impaired glucose homeostasis are closely related, and during the last decades of investigation about vitamin D, several clinical and epidemiological studies documented an inverse correlation between circulating vitamin D levels, central adiposity and the development of insulin resistance and diabetes. The insufficient sun exposure and outdoor activities of obese individuals, the storage of vitamin D in adipose tissue, because of its lipophilic properties, and the vitamin D-mediated modulation of adipogenesis, insulin secretion, insulin sensitivity and the immune system, are the main reasons for the close relationship between obesity, glucose homeostasis and hypovitaminosis D. Then objective of this review is to explore the pathophysiological mechanism(s) by which vitamin D modulates glycemic control and insulin sensitivity in obese individuals.

## 1. Introduction

Type 2 diabetes (T2DM) and obesity are both rapidly increased during the last decades and are continuing to increase at an alarming rate worldwide. The World Health Organization recently stated that 90% of all cases of diabetes refer to T2DM, and that worldwide about 15 million subjects are affected by T2DM, and this number might be doubled by 2025 [1].

Obesity and T2DM are closely related, since some altered obesity-induced conditions, such as excess of fat mass, systemic low-grade inflammation, altered insulin intracellular signaling and pancreatic β cells dysfunction, are involved in both insulin resistance and T2DM development [1,2].

Based on recent evidence, hypovitaminosis D seems to play a part in the pathogenesis of a large number of metabolic diseases, both in children and adults, and in particular an association between vitamin D deficiency and insulin resistance was proposed, as well as a potential role of hypovitaminosis D in the development of T2DM in obese subjects [1,2,3]. In fact, in addition to the recognized calciotropic effects, vitamin D is essential for the modulation/regulation of the immune system, pancreas, liver, skeletal muscle and adipocytes (Table 1), and several clinical studies demonstrated that vitamin D supplementation in subjects with T2DM and metabolic syndrome improves lipid profile, glycated hemoglobin (HbA1c) and insulin sensitivity (HOMA-IR) [1]. 

Although the association between hypovitaminosis D and obesity-induced metabolic disorders is well known, to date the mechanisms of this relationship are not completely clarified. The objectives of this manuscript are to revise the studies on the association between vitamin D status and obesity and obesity-induced insulin resistance, and to explore the pathophysiological mechanism(s) by which vitamin D modulates insulin sensitivity and glycemic control in obese individuals. VDR: vitamin D receptor.

## 2. Vitamin D Deficiency and Obesity

Obesity is a pathological state characterized by an excessive body weight due to an accumulation of fat mass, caused by a food addiction, genetic mutations, sedentary life style, endocrine disorders and poor nutritional status. It is universally accepted that obesity represents a worldwide health problem since it is associated with comorbidities, such as metabolic syndrome, diabetes, hypertension, pulmonary diseases, non-alcoholic fatty liver disease, renal disease, cardiovascular disease, skeletal alterations and cancer. Moreover, obesity is also often linked with low levels of serum vitamins, including vitamin B1 (thiamine), folate and vitamin D [4,23,24]. 

During the last 3 decades of investigation regarding vitamin D, including its metabolism and its skeletal and extra-skeletal functions, several epidemiological and clinical studies have documented an inverse correlation between circulating vitamin D levels, central adiposity and the development of obesity [25,26,27], as well as an association among low levels of circulating 25-hydroxyvitamin D (25(OH)D), obesity and metabolic syndrome, both in children and adults, and regardless of ethnicity and geographical area [18,28,29,30,31]. Moreover, some studies demonstrated that the association between obesity and hypovitaminosis D is independent of vitamin D supplementation and dietetic differences, and have concluded that the risk of vitamin D deficiency is amplified in overweight and obese individuals more by the excess adiposity than insufficient intake [29,30,31,32]. However, up to now, the correlation between poor vitamin D status and several variables, such as high fat mass, sun exposure, food intake and exercise, have not been precisely determined. Probably, in obese subjects, the insufficient sun exposure and outdoor activities and the altered expression of the vitamin D receptor (VDR) among adipose tissue could represent the principal factors of the association between obesity and vitamin D deficiency [33,34,35].

The genomic responses to vitamin D result from the stereospecific interactions between its active metabolite (1,25(OH)_2_D) with its intracellular VDR, a member of the superfamily of the steroid hormone receptors. Then, after binding to the hormone, VDR forms a heterodimer with the retinoid-X receptor (RXR) that binds to specific vitamin D response elements (VDREs) on DNA sequences leading to expression or trans-repression of some gene products [36]. Indeed, VDR modulates the expression of several genes, which regulate calcium/phosphate homeostasis, cellular proliferation and differentiation and immune response [37], and modulates glucose tolerance and insulin sensitivity since it is expressed by pancreatic β-cells [37,38,39], adipose tissue [40] and skeletal muscle [41]. 

Adipogenesis is the process by which different events lead to the differentiation of preadipocytes in mature adipocytes and, interestingly, hypertrophy of adipocytes is the cause of increased adiposity, leading to obesity. In vitro experiments performed 30 years ago demonstrated that vitamin D could inhibit adipogenesis and that triglyceride accumulation was blunted by 50% in 3T3-L1 preadipocytes exposed to 1,25(OH)_2_D, compared to the unexposed cells [5,6]. Subsequent studies reported that *Cyp27b1,* the gene that encodes the enzyme converting 25(OH)D to 1,25(OH)_2_D, is expressed in fat tissue of rodents and humans [7], and that vitamin D inhibits adipogenesis through a mechanism involving the peroxisome proliferator-activated receptor gamma (PPARγ) and VDR in a competitive manner [4]. PPARγ shares a mutual heterodimeric binding partner, RXR, with VDR, and an increased expression of VDR correlates with a reduction in PPARγ-induced adipogenesis and with a decreased mitotic clonal adipocyte population [4]. Moreover, a study on porcine derived preadipocytes has shown that 1,25(OH)_2_D inhibits cell differentiation and the expression of PPARγ and other adipocyte marker genes (Lpl, Pck2, Scd) [8], and a study conducted on murine mesenchymal cells treated with 18 × 10^12^ mol/L vitamin D reported decreased differentiation of adipocytes from bone marrow derived-cells [9].

VDR is also implicated in the regulation of thermogenesis, since it directly controls the uncoupling protein 1 (UCP1) and uncoupling protein 2 (UCP2). In particular, the expression of UCP2 in human adipocytes, which seems to play a role in the pathogenetic mechanisms of insulin resistance and diabetes development, is significantly blunted by 1,25(OH)_2_D through the nuclear VDR activation [10]. Moreover, a clinical study reported a decrease in body weight and fat mass by a low energy diet and vitamin D supplementation [42,43], while in an in vivo murine model, vitamin D supplementation prevented animals from diet induced obesity [44].

Conversely, fat mass represents a reservoir of vitamin D, which is a fat-soluble compound. Vitamin D can accumulate in the body, where it is spread widely, but it is mainly stored in fat tissue and then gradually released. In obese subjects, the enlarged pool of visceral and subcutaneous adipose tissue probably impounds vitamin D and its metabolites, reducing their bioavailability. In fact, in obese people undergoing bariatric surgery, it was demonstrated that an inverse concentration of 25(OH)D was measured in serum and within subcutaneous adipose tissue, showing, respectively, a negative and positive association with body weight and adiposity [45]. 

Finally, it has been reported that obese individuals have a reduced extent of sunlight exposure due to lower amount of physical activity and mobility, and also often because of psychological distress [46].

## 3. Vitamin D Deficiency, Insulin Resistance and Diabetes

The deregulation of insulin signaling pathways is the main factor causing reduced insulin sensitivity and then insulin resistance, and insulin resistance is the most frequent cause for T2DM [47]. Increasing evidence suggests that an altered insulin sensitivity, in both humans and experimental animal models, strongly correlates with obesity and hypovitaminosis D. Moreover, it has been established that vitamin D deficiency leads to the development of insulin resistance and T2DM, albeit the biological mechanisms are not fully understood.

The proposed mechanisms by which vitamin D modulates glycemic homeostasis also involve modulation of glucose-mediated synthesis/secretion of insulin by β-cells, increasing both hepatic and peripheral glucose uptake by direct and indirect mechanisms, and blunting inflammation [48,49,50,51]. 

VDR and the 1-α-hydroxylase enzyme are expressed in pancreatic β-cells, and it has been reported that 1,25(OH)_2_D may have a role in the regulation of insulin production and secretion [52]; in particular, it has been demonstrated that 1,25(OH)_2_D acts on pancreatic islets stressed by inflammation and vitamin D deficiency [53]. Moreover, it is well established that normal levels of vitamin D are essential for keeping extracellular calcium concentrations and calcium influx into β-cells for insulin secretion, while VDR signaling might play a direct role in glucose-induced insulin secretion [11]. 

In addition to regulating insulin synthesis and secretion, VDR signaling promotes insulin-induced glucose uptake in liver, adipose and skeletal muscle tissues [12], and 1,25(OH)_2_D directly activates the transcription and expression of the insulin receptor gene and protein [13,14,15] in both humans and experimental animal models. Further, it has been also reported that, in vivo, 1,25(OH)_2_D upregulates the expression of GLUT-4 in muscle cells and promotes its translocation in animal model adipocytes [16,17].

Another important factor that closely links obesity and insulin resistance is the low-grade chronic inflammation that often is observed in obese subjects as a direct consequence of an amplified production of pro-inflammatory cytokines by macrophages and adipocytes [27]. Vitamin D modulates the immune system, and several studies have demonstrated that, in the presence of central adiposity, vitamin D deficiency correlates with inflammation and reduced insulin sensitivity [27,54], while vitamin D supplementation improves them [55,56,57]. Vitamin D induces a lower chemokine and cytokine release by adipocytes and the chemotaxis of monocytes, and its effects on systemic and tissue-specific inflammation have been ascribed to multiple factors, including suppressing the NF-κβ pathway, shifting T-helper cells towards the anti-inflammatory TH2 subset, blunting the expression of toll-like receptor 4 (TLR-4) and reducing the differentiation of dendritic cells [19,20,21,58,59].

Finally, the adipokine adiponectin, which is secreted by fat tissue in an opposite ratio to the body weight and the body mass index, might represent a potential link between vitamin D deficiency and insulin resistance, since low circulating levels of both are associated with impaired insulin sensitivity, independent of the degree of adiposity [22,60]. Adiponectin has been shown to have an insulin-sensitizing effect in peripheral tissues, as well as modulatory effects on gluconeogenesis, and its receptors are also expressed in pancreatic β-cells [61,62]. How vitamin D interacts with adiponectin is not well understood. Probably they are linked because adiponectin and glucose metabolism are regulated by osteocalcin, an osteoblast-derived protein, which in turn is influenced by vitamin D, and also because 1,25(OH)_2_D modulates adipogenesis through a VDR-dependent mechanism [63,64], interacting with PPARγ [60,65,66].

## 4. Effect of Vitamin D Supplementation on Glucose Tolerance 

As stated above, during the last 30 years of investigation on vitamin D, several clinical studies have documented an inverse correlation between circulating vitamin D levels, central adiposity and the development of obesity, metabolic syndrome and glucose homeostasis impairment, both in children and adults [18,25,26,27,28,29,30,31]. Obviously, to confirm these clinical observations, it is necessary to further explore the effects of vitamin D supplementation on glucose metabolic derangements, in obese, vitamin-D deficient individuals.

Many intervention studies have demonstrated that the replacement of serum 25(OH)D levels in subjects with vitamin D deficiency can improve glucose tolerance in centrally obese but non-diabetic individuals [67,68], and that this effect was dependent on the dose and duration of administration [69,70]. Moreover, in a subsequent study, in which subjects affected by type 2 diabetes were administered calcitriol 0.5 mcg/daily for 12 weeks, an increase in insulin secretion was observed, but no effects on modulation of insulin resistance were seen [71]. Further, studies in which calcium and vitamin D were administered in adult or aged subjects depicted a reduced risk of T2DM and an improvement of plasma glucose and insulin resistance in both healthy subjects and individuals affected by metabolic syndrome [25,72,73].

In contrast, studies in which calcium and vitamin D were administered chronically for 7 years, as in the Women’s Health Initiative intervention trial [74] or in osteoporotic fractures prevention trial [75], did not show a protective outcome against the development of T2DM. The supplementation with high dosages of vitamin D before an oral glucose tolerance test (OGTT) did not report effects on plasma glucose and insulin secretion [76], and the supplementation with vitamin D for 6 months in diabetic individuals with normal vitamin D status did not induce any improvement in relationships to glycated hemoglobin levels, insulin secretion or resistance [77].

Finally, only a few intervention studies concerning vitamin D supplementation for obese adolescents have been performed. Two of these were comparable in length (12 weeks) and in study population characteristics, and both studies confirmed positive effects of vitamin D supplementation on glucose homeostasis and/or metabolic syndrome outcomes [78,79]. However, a subsequent study, on non-diabetic obese adolescents with good vitamin D status, reported that vitamin D supplementation, independent of its dose, did not demonstrate effects on β-cell function or insulin action, while for obese adolescents with hypovitaminosis D and impaired glucose metabolism, vitamin D supplementation determined heterogeneous responses on insulin modulation, which, however, still remain unclear [80]. Further, a recently published large clinical intervention study demonstrated that vitamin D3 supplementation at a dose of 4000 IU per day did not significantly lower the risk of diabetes as compared to placebo [81].

## 5. Conclusions

Vitamin D deficit is strongly linked with obesity, and it is involved in the development of insulin resistance and T2DM. The main reasons of this close relationship are mostly due to the storage of vitamin D in adipose tissue, due to its lipophilic properties, and to its direct action on adipogenesis, regulation of insulin secretion, modulation of insulin sensitivity in peripheral tissues and modulation of immune system.

The treatment of hyperglycemia, or impaired glucose tolerance due to insulin resistance that associated with obesity, is based on lifestyle intervention, with nutritional and physical activity measurements, and even hypoglycemic drugs.

Due to the close link existing between obesity, glucose homeostasis and hypovitaminosis D, it might be desirable to have a good vitamin D status, and grounded on the known mechanism(s) of action of vitamin D, obese individuals might represent the main recipients of the effects of vitamin D on the modulation of insulin sensitivity and T2DM prevention. 

However, nowadays, the data from intervention studies with vitamin D supplementation to recover insulin resistance and glucose tolerance are still controversial, both in obese children and adults. Likely, the main reasons of these conflicting results are due to the diverse populations studied, biochemical preparations of vitamin D used, doses and length of supplementation. Then, there is the need for additional randomized controlled trials concentrating on obese subjects with recognized vitamin D deficit and the cautious choice of the dose, dosing schedule and attainment of target 25(OH)D serum level. The trials should also comprise clamp measurements of the in vivo β-cell sensitivity and function to fully depict and understand the effects of vitamin D replacement on insulin resistance. 

## Figures and Tables

**Table 1:** Principal biological mechanisms by which vitamin D modulates adipogenesis and glucose homeostasis in humans, animal and cellular models.

1,25(OH)_2_D inhibits adipogenesis and reduces triglyceride accumulation in 3T3-L1 preadipocytes.	[4,5,6,7]
1,25(OH)_2_D inhibits cell differentiation, the expression of PPAR and other adipocyte marker genes (Lpl, Pck2, Scd) in porcine derived preadipocytes.	[8]
Vitamin D reduces murine mesenchymal cells differentiation into adipocytes.	[9]
1,25(OH)_2_D suppresses the expression of UCP2 in human adipocytes through the nuclear VDR activation.	[10]
Vitamin D is essential in maintaining extracellular calcium concentrations and calcium influx into β-cells for insulin secretion; VDR signaling may play a direct role in glucose-induced insulin secretion.	[11]
VDR signaling promotes insulin-stimulated glucose uptake in skeletal muscle, adipose tissue and liver.	[12]
1,25(OH)_2_D directly activates the transcription of insulin receptor gene and increases the expression of the insulin receptor, both in humans and animal models.	[13,14,15]
1,25(OH)_2_D upregulates the expression of GLUT-4 in skeletal muscle and promotes its translocation in animal model adipocytes.	[16,17]
Vitamin D inhibits the NF-κβ pathway, shifting T-helper cells towards the anti-inflammatory TH2 subset; decreases the expression of toll-like receptor 4 (TLR-4); decreases the maturation of dendritic cells.	[18,19,20,21,22]

## References

[1] Pajor I.S., Sliwinska A. (2019). Analysis of association between vitamin D deficiency and insulin resistance. Nutrients..

[2] Wang H., Chen W., Li D., Yin X., Zhang X., Olsen N., Zheng S.G. (2017). Vitamin D and Chronic Diseases. Aging Dis..

[3] Li Y.-X., Zhou L. (2015). Vitamin D Deficiency, Obesity and Diabetes. Cell. Mol. Biol. Noisy.

[4] Wood R.J. (2008). Vitamin D and adipogenesis: new molecular insights. Nutr. Rev..

[5] Ishida Y., Taniguchi H., Baba S. (1988). Possible involvement of 1 alpha,25- dihydroxyvitamin D3 in proliferation and differentiation of 3T3-L1 cells. Biochem. Biophys. Res. Commu..

[6] Sato M., Hiragun A. (1988). Demonstration of 1alpha,25-dihydroxyvitamin D3 receptor-like molecule in ST 13 and 3T3 L1 pre-adipocytes and its inhibitory effects on preadipocyte differentiation. J. Cell Physiol..

[7] Li J., Byrne M.E., Chang E., Jiang Y., Donkin S.S., Buhman K.K., Burgess J.R., Teegarden D. (2008). 1Alpha,25-dihydroxyvitamin D hydroxylase in adipocytes. J. Steroid. Biochem. Mol. Biol..

[8] Zhuang H., Lin Y., Yang G. (2007). Effects of 1,25-dihydroxyvitamin D3 on proliferation and differentiation of porcine preadipocyte in vitro. Chem. Biol. Interact..

[9] Duque G., Macoritto M., Kremer R. (2004). 1,25(OH)2D3 inhibits bone marrow adipogenesis in senescence accelerated mice (SAM-P/6) by decreasing the expression of peroxisome proiferator-activated receptor gamma 2 (PPARgamma2). Exp. Gerontol..

[10] Shi H., Norman A.W., Okamura W.H., Sen A., Zemel M.B. (2002). 1Alpha,25-dihydroxyvitamin D3 inhibits uncoupling protein 2 expression in human adipocytes. FASEB J..

[11] Lee S., Clark S.A., Gill R.K., Christakos S. (1994). 1,25-Dihydroxyvitamin D3 and pancreatic beta-cellfunction: Vitamin D receptors, gene expression, and insulin secretion. Endocrinology.

[12] Huang Y., Ishizuka T., Miura A., Kajita K., Ishizawa M., Kimura M., Yamamoto Y., Kawai Y., Morita H., Uno Y. (2002). Effect of 1 alpha,25-dihydroxy vitamin D3 and vitamin E on insulin-induced glucose uptake in rat adipocytes. Diabetes Res. Clin. Pract..

[13] Calle C., Maestro B., Garcia-Arencibia M. (2008). Genomic actions of 1,25-dihydroxyvitamin D3 on insulin receptor gene expression, insulin receptor number and insulin activity in the kidney, liver and adipose tissue of streptozotocin-induced diabetic rats. BMC Mol. Biol..

[14] Maestro B., Davila N., Carranza M., Calle C. (2003). Identification of a vitamin D response element in the human insulin receptor gene promoter. J. Steroid Biochem Mol. Biol..

[15] Maestro B., Molero S., Bajo S., Davila N., Calle C. (2002). Transcriptional activation of the human insulin receptor gene by 1,25-dihydroxyvitamin D(3). Cell Biochem Funct..

[16] Castro A., Frederico M., Cazarolli L., Bretanha L., Tavares L., Buss Z., Dutra M.F., de Souza A.Z., Pizzolatti M.G., Silva F.R. (2014). Betulinic acid and 1,25(OH)(2) vitamin D(3) share intracellular signal transduction in glucose homeostasis in soleus muscle. Int. J. Biochem. Cell Biol..

[17] Manna P., Jain S. (2012). Vitamin D up-regulates glucose transporter 4 (GLUT4) translocation and glucose utilization mediated by cystathionine-gamma-lyase (CSE) activation and H2S formation in 3T3L1 adipocytes. J. Biol. Chem..

[18] Holick M.F. (2011). Vitamin D: Evolutionary, physiological and health perspectives. Curr. Drug. Targets.

[19] Chen Y., Zhang J., Ge X., Du J., Deb D., Li Y. (2013). Vitamin D receptor inhibits nuclear factor kappa-B activation by interacting with I-kappa-B kinase beta protein. J. Biol. Chem..

[20] Ding C., Wilding J., Bing C. (2013). 1,25-dihydroxyvitamin D3 protects against macrophage-induced activation of NF-kappa-B and MAPK signalling and chemokine release in human adipocytes. PLoS ONE.

[21] Guillot X., Semerano L., Saidenberg-Kermanac’h N., Falgarone G., Boissier M. (2010). Vitamin D and inflammation. Joint Bone Spine..

[22] Whitehead J.P., Richards A.A., Hickman I.J., Macdonald G.A., Prins J.B. (2006). Adiponectin–a key adipokine in the metabolic syndrome. Diabetes Obes. Metab..

[23] Kant A.K. (2003). Interaction of body mass index and attempt to lose weight in a national sample of US adults: Association with reported food and nutrient intake, and biomarkers. Eur J. Clin. Nutr..

[24] Kerns J.C., Arundel C., Chawla L.S. (2015). Thiamin deficiency in people with obesity. Adv. Nutr..

[25] Liu S., Song Y., Ford E.S., Manson J.E., Buring J.E., Ridker P.M. (2005). Dietary calcium, vitamin D, and the prevalence of metabolic syndrome in middle-aged and older U.S. women. Diabetes Care.

[26] Ford E.S., Ajani U.A., McGuire L.C., Liu S. (2005). Concentrations of serum vitamin D and the metabolic syndrome among U.S. adults. Diabetes Care.

[27] Fornari R., Francomano D., Greco E.A., Marocco C., Lubrano C., Wannenes F., Papa V., Bimonte V.M., Donini L.M., Lenzi A. (2015). Lean mass in obese adult subjects correlates with higher levels of vitamin D, insulin sensitivity and lower inflammation. J. Endocrinol. Invest..

[28] Botella-Carretero J.I., Alvarez-Blasco F., Villafruela J.J., Balsa J.A., Vázquez C., Escobar-Morreale H.F. (2007). Vitamin D deficiency is associated with the metabolic syndrome in morbid obesity. Clin. Nutr..

[29] Greene-Finestone L.S., Garriguet D., Brooks S., Langlois K., Whitng S.J. (2017). Overweight and obesity are associated with lower vitamin D status in Canadian children and adolescents. Paediatr. Child. Health..

[30] Mezza T., Muscogiuri G., Sorice G.P., Prioletta A., Salomone E., Pontecorvi A., Giaccari A. (2012). Vitamin D deficiency: A new risk factor for type 2 diabetes?. Ann. Nutr. Metab..

[31] Saneei P., Salehi-Abargouei A., Esmaillzadeh A. (2013). Serum 25-hy- droxy vitamin D levels in relation to body mass index: A systematic review and meta-analysis. Obes. Rev..

[32] Rodríguez-Rodríguez E., Navia B., López-Sobaler A.M., Ortega R.M. (2009). Vitamin D in overweight/obese women and its relationship with dietetic and anthropometric variables. Obesity.

[33] Pereira-Santos M., Costa P.R., Assis A.M., Santos C.A., Santos D.B. (2015). Obesity and vitamin D deficiency: A systematic review and meta-analysis. Obes. Rev..

[34] Wong K.E., Kong J., Zhang W., Szeto F.L., Ye H., Deb D.K., Brady M.J., Li Y.C. (2011). Targeted expression of human vitamin D receptor in adipocytes decreases energy expenditure and induces obesity in mice. J. Biol Chem..

[35] Kumar J., Muntner P., Kaskel F.J., Hailpern S.M., Melamed M.L. (2009). Prevalence and associations of 25hydroxyvitamin D deficiency in US children: NHANES 2001–2004. Pediatrics.

[36] Haussler M., Whitfield G., Kaneko I., Haussler C., Hsieh D., Hsieh J., Jurutka P.W. (2013). Molecular mechanisms of vitamin D action. Calcif. Tissue Int..

[37] Wang Y., Zhu J., De Luca H. (2012). Where is the vitamin D receptor?. Arch. Biochem. Biophys..

[38] Mitri J., Dawson-Hughes B., Hu F., Pittas A. (2011). Effects of vitamin D and calcium supplementation on pancreatic beta cell function, insulin sensitivity, and glycemia in adults at high risk of diabetes: The Calcium and Vitamin D for Diabetes Mellitus (CaDDM) randomized controlled trial. Am. J. Clin. Nutr..

[39] Zeitz U., Weber K., Soegiarto D., Wolf E., Balling R., Erben R. (2003). Impaired insulin secretory capacityin mice lacking a functional vitamin D receptor. FASEB J..

[40] Ding C., Gao D., Wilding J., Trayhurn P., Bing C. (2012). Vitamin D signalling in adipose tissue. Br. J. Nutr..

[41] Ceglia L. (2009). Vitamin D and its role in skeletal muscle. Curr. Opin. Clin. Nutr. Metab. Care..

[42] Raederstorff D. (2009). Antioxidant activity of olive polyphenols in humans: A review. Int. J. Vitam. Nutr. Res..

[43] Zhu W., Cai D., Wang Y., Lin N., Hu Q., Qi Y., Ma S., Amarasekara S. (2013). Calcium plus vitamin D3 supplementation facilitated fat loss in overweight and obese college students with very-low calcium consumption: A randomized controlled trial. Nutr. J..

[44] Sergeev I.N., Song Q. (2014). High vitamin D and calcium intakes reduce diet-induced obesity in mice by increasing adipose tissue apoptosis. Mol. Nutr. Food Res..

[45] Blum M., Dolnikowski G., Seyoum E., Harris S.S., Booth S.L., Peterson J., Saltzman E., Dawson-Hughes B. (2008). Vitamin D3 in fat tissue. Endocrine.

[46] Compston J.E., Vedi S., Ledger J.E., Webb A., Gazet J.C., Pilkington T.R. (1981). Vitamin D status and bone histomorphometry in gross obesity. Am. J. Clin. Nutr..

[47] Ozougwu J.C., Obimba K.C., Belonwu C.D., Unakalamba C.B. (2013). The pathogenesis and pathophysiology of type 1 and type 2 diabetes mellitus. J. Physiol. Pathophysiol..

[48] Martin T., Campbell R.K. (2011). Vitamin D and diabetes. Diabetes Spectr..

[49] Gagnon C., Daly R.M., Carpentier A., Lu Z.X., Shore-Lorenti C., Sikaris K., Jean S., Ebeling P.R. (2014). Effects of combined calcium and vitamin D supplementation on insulin secretion, insulin sensitivity and β-cell function in multi-ethnic vitamin D-deficient adults at risk for type 2 diabetes: A pilot randomized, placebo-controlled trial. PLoS ONE.

[50] Gao Y., Wu X., Fu Q., Li Y., Yang T., Tang W. (2015). The relationship between serum 25-hydroxy vitamin D and insulin sensitivity and β-cell function in newly diagnosed type 2 diabetes. J. Diabetes Res..

[51] Park S., Kim D.S., Kang S. (2016). Vitamin D deficiency impairs glucose-stimulated insulin secretion and increases insulin resistance by reducing PPAR-γ expression in nonobese Type 2 diabetic rats. J. Nutr. Biochem..

[52] Billaudel B., Delbancut A., Sutter B., Faure A. (1993). Stimulatory effect of 1,25-dihydroxyvitamin D3 on calcium handling and insulin secretion by islets from vitamin D3-deficient rats. Steroids.

[53] Wolden-Kirk H., Overbergh L., Gysemans C., Brusgaard K., Naamane N., Van Lommel L., Schuit F., Eizirik D.L., Christesen H., Mathieu C. (2013). Unraveling the effects of 1,25OH2D3 on global gene expression in pancreatic islets. J. Steroid. Biochem. Mol. Biol..

[54] Flores M. (2005). A role of vitamin D in low-intensity chronic inflammation and insulin resistance in type 2diabetes mellitus?. Nutr. Res. Rev..

[55] Hopkins M., Owen J., Ahearn T., Fedirko V., Flanders W., Jones D., Bostick R.M. (2011). Effects of supplemental vitamin D and calcium on biomarkers of inflammation in colorectal adenoma patients: A randomized, controlled clinical trial. Cancer Prev. Res..

[56] Shab-Bidar S., Neyestani T., Djazayery A., Eshraghian M., Houshiarrad A., Kalayi A., Shariatzadeh N., Khalaji N., Gharavi A. (2012). Improvement of vitamin D status resulted in amelioration of biomarkers of systemic inflammation in the subjects with type 2 diabetes. Diabetes Metab. Res. Rev..

[57] Wamberg L., Cullberg K., Rejnmark L., Richelsen B., Pedersen S. (2013). Investigations of the anti-inflammatory effects of vitamin D in adipose tissue: Results from an *in vitro* study and a randomized controlled trial. Horm. Metab. Res..

[58] Cantorna M., Zhu Y., Froicu M., Wittke A. (2004). Vitamin D status, 1,25-dihydroxyvitamin D3, and the immune system. Am. J. Clin. Nutr..

[59] Du T., Zhou Z., You S., Lin J., Yang L., Zhou W.D., Huang G., Chao C. (2009). Regulation by 1, 25-dihydroxy- vitamin D3 on altered TLRs expression and response to ligands of monocyte from autoimmune diabetes. Clin. Chim. Acta..

[60] Nimitphong H., Chanprasertyothin S., Jong-Jaroenprasert W., Ongphiphadhanakul B. (2009). The association between vitamin D status and circulating adiponectin independent of adiposity in subjects with abnormal glucose tolerance. Endocrine.

[61] Berg A., Combs T., Du X., Brownlee M., Scherer P. (2001). The adipocyte-secreted protein Acrp30 enhances hepatic insulin action. Nat. Med..

[62] Park S., Kim D., Kwon D., Yang H. (2011). Long-term central infusion of adiponectin improves energy and glucose homeostasis by decreasing fat storage and suppressing hepatic gluconeogenesis without changing food intake. J. Neuroendocrinol..

[63] Lee N.K., Sowa H., Hinoi E., Ferron M., Ahn J.D., Confavreux C., Dacquin R., Mee P.J., McKee M.D., Jung D.Y. (2007). Endocrine regulation of energy metabolism by the skeleton. Cell.

[64] Carvallo L., Henriquez B., Olate J., van Wijnen A.J., Lian J.B., Stein G.S., Onate S., Stein J.L., Mon- tecino M. (2007). The 1alpha,25-dihydroxy vitamin D3 receptor preferentially recruits the coactivator SRC-1 during up-regulation of the osteocalcin gene. J. Steroid. Biochem. Mol. Biol..

[65] Liu E., Meigs J., Pittas A., McKeown N., Economos C., Booth S., Jacques P.F. (2009). Plasma 25-hydroxyvitamin D is associated with markers of the insulin resistant phenotype in nondiabetic adults. J. Nutr..

[66] Maeda N., Takahashi M., Funahashi T., Kihara S., Nishizawa H., Kishida K., Nagaretani H., Matsuda M., Komuro R., Ouchi N. (2001). PPAR-gamma ligands increase expression and plasma concentrations of adiponectin, an adipose-derived protein. Diabetes.

[67] Kumar S., Davies M., Zakaria Y., Mawer E.B., Gordon C., Olukoga A.O., Boulton A.J. (1994). Improvement in glucose tolerance and beta-cell function in a patient with vitamin D deficiency during treatment with vitamin D. Postgrad. Med. J..

[68] Nagpal J., Pande J.N., Bhartia A. (2009). A double-blind, randomized, placebo-controlled trial of the short-term effect of vitamin D3 supplementation on insulin sensitivity in apparently healthy, middle-aged, centrally obese men. Diabet. Med..

[69] von Hurst P.R., Stonehouse W., Coad J. (2010). Vitamin D supplementation reduces insulin resistance in South Asian women living in New Zealand who are insulin resistant and vitamin D deficient–a randomised, placebo-controlled trial. Br. J. Nutr..

[70] Vieth R., Bischoff-Ferrari H., Boucher B.J., Dawson-Hughes B., Garland C.F., Heaney R.P., Holick M.F., Hollis B.W., Lamberg-Allardt C., McGrath J.J. (2007). The urgent need to recommend an intake of vitamin D that is effective. Am. J. Clin. Nutr..

[71] Akbarzadeh M., Dabbaghmanesh M.H., Hasanzadeh J., Hasanzadeh J. (2011). Impact of treatment with oral calcitriol on glucose indices in type 2 diabetes mellitus patients. J. Clin. Nutr..

[72] Pittas A.G., Dawson-Hughes B., Li T., Van Dam R.M., Willett W.C., Manson J.E., Hu F.B. (2006). Vitamin D and calcium intake in relation to type 2 diabetes in women. Diabetes Care.

[73] Pittas A.G., Harris S.S., Stark P.C., Dawson-Hughes B. (2007). The effects of calcium and vitamin D supplementation on blood glucose and markers of inflammation in nondiabetic adults. Diabetes Care.

[74] de Boer I.H., Tinker L.F., Connelly S., Curb J.D., Howard B.V., Kestenbaum B., Larson J.C., Manson J.E., Margolis K.L., Siscovick D.S. (2008). Women’s Health Initiative Investigators: Calcium plus vitamin D supplementation and the risk of incident diabetes in the Women’s Health Initiative. Diabetes Care.

[75] Grant A.M., Avenell A., Campbell M.K., Mc-Donald A.M., MacLennan G.S., McPherson G.C., Anderson F.H., Cooper C., Francis R.M., Donaldson C. (2005). RECORD Trial Group: Oral vitamin D3 and calcium for secondary prevention of low-trauma fractures in elderly people (Randomised Evaluation of Calcium Or vitamin D, RECORD): A randomised placebo-controlled trial. Lancet.

[76] Tai K., Need A.G., Horowitz M., Chapman I.M. (2008). Glucose tolerance and vitamin D: Effects of treating vitamin D deficiency. Nutrition.

[77] Jorde R., Figenschau Y. (2009). Supplementation with cholecalciferol does not improve glycaemic control in diabetic subjects with normal serum 25-hydroxyvitamin D levels. Eur. J. Nutr..

[78] Belenchia A., Tosh A., Hillman L., Peterson C. (2013). Correcting vitamin D insufficiency improves insulin sensitivity in obese adolescents: A randomized controlled trial. Am. J. Clin. Nutr..

[79] Kelishadi R., Salek S., Salek M., Hashemipour M., Movahedian M. (2014). Effects of vitamin d supplementation on insulin resistance and cardiometabolic risk factors in children with metabolic syndrome: A trolpe-masked controlled trial. J. Pediatr..

[80] Javed A., Vella A., Balagopal P.B., Fischer P.R., Weaver A.L., Piccinini F., Dalla Man C., Cobelli C., Giesler P.D., Laugen J.M. (2015). Cholecalciferol supplementation does not influence β-cell functionand insulin action in obese adolescents: A prospective double-blind randomized trial. J. Nutr..

[81] Pittas A.G., Dawson-Hughes B., Sheehan P., Ware J.H., Knowler W.C., Aroda V.R., Brodsky I., Ceglia L., Chadha C., Chatterjee R. D2d Research Group. Vitamin D Supplementation and Prevention of Type 2 Diabetes. N. Engl. J. Med..

